# Quality of life in allergic rhinitis - children’s and their parents’ perspective in polish urban and rural population

**DOI:** 10.1186/s12955-020-01315-1

**Published:** 2020-03-10

**Authors:** Hanna Sikorska-Szaflik, Barbara Sozańska

**Affiliations:** 1st Department and Clinic of Paediatrics, Allergology and Cardiology, Wroclaw Medical University, ul. Chalubinskiego 2a, 50-368 Wroclaw, Poland

**Keywords:** Allergic rhinitis, Quality of life, Children, T4SS, VAS, KINDL-R questionnaire

## Abstract

**Background:**

Allergic rhinitis is a common chronic condition in the paediatric population. No reports regarding the quality of life in children with allergic rhinitis in the Polish population have been found in the available literature. The aim of this study was to assess and compare the quality of life in patients with allergic rhinitis reported by children and their parents living in a city and in rural areas, and to evaluate the possible relationships between the quality of life and the severity of symptoms.

**Methods:**

Two hundred and eight children with allergic rhinitis participated in the study (89 girls, aged 6–17, mean age 11.7 ± 3). Children were asked to evaluate their rhinitis symptoms by using two scales: the *Total 4 Symptom Score* and the *Visual Analogue Scale.* The quality of life assessment included the KINDL-R questionnaire.

**Results:**

Both for the T4SS and the VAS scale the severity of symptoms in children with seasonal rhinitis was significantly higher than in children allergic to perennial allergens. The quality of life total scores on the KINDL questionnaire was 45.6 ± 8.5 for the children and 73.7 ± 10.7 for the parents. In all the domains, except for physical health, the child’s quality of life was rated significantly higher by parents than by children. The biggest discrepancy occurred in the domains: social contacts and family.

**Conclusions:**

Allergic rhinitis can disrupt the quality of life. Parents tend to overestimate their children’s quality of life comparing to the children’s own assessment. The quality of life in children with allergic rhinitis correlated with the severity of the clinical symptoms of the disease. Evaluation of the quality of life in children is an essential issue in clinical investigation of patients with allergic rhinitis. It is of great importance to ask children themselves about their quality of life than rely only on parental opinion.

## Background

Allergic rhinitis (AR) is a common chronic condition in the paediatric population. The prevalence of AR in children is high and still progressively increasing [1]. It is estimated to occur in between 4.2 and 12.7% of children aged 6–7, and 1 to 45.1% of 13 and 14-year-olds worldwide [2]. In Poland, according to the results of the ECAP (Epidemiology of Allergic Diseases in Poland) survey, AR diagnosed by a physician was present in 24.4% of children aged 6–7, and in 30.9% of adolescents aged 13–14. There is also a problem with underdiagnosis of the disease due to the fact that adolescents frequently do not report symptoms of the illness to the doctor, and undergo symptomatic treatment without consulting a specialist [3].

In the recent years, evaluation of the quality of life (QoL) has been considered an important issue in clinical investigation. The available literature provides no reports regarding the QoL in children with AR in the Polish population. Nevertheless, AR has a noticeable impact on the QoL and impairs daily activities. Patients may also suffer from sleep disorders, emotional problems, impairment in activities and social functioning [4]. Children with AR may have difficulties at school related to fatigue, irritability, and sleep disorders occurring due to nasal obstruction [5, 6]. Social contact with parents and peers may also be negatively affected by the AR symptoms. Because of the rhinorrhea, nasal congestion, and frequent sneezing, participation in social and family meetings might pose a problem for children with AR. This results in emotional disorders and leads to sadness, anger, frustration and withdrawal [4]. An important aspect of the child’s QoL assessment is the possibility to ask children directly, and then confront their perspective with the parent’s views. In the study carried out on healthy children, it has been shown that carers tend to overestimate their children’s QoL compared to the children’s own assessment [7]. Apart from the data from the parents’ report, it is also essential to relate to children’s own opinion about their well-being. Interestingly, the place of residence can affect the QoL. According to some studies, children living in the country have higher QoL compared to their city peers [8, 9].

The aim of this study was to assess and compare the QoL in patients with AR, reported by children and their parents living in a city and in rural areas, and to evaluate the possible relationships between the QoL and the severity of symptoms.

## Methods

We obtained approval for the survey from the Ethics Committee at the Wroclaw Medical University. Parents and children received detailed information regarding the background and the implementation of the study, and were given the opportunity to withdraw their children from participation at any time. Each participant had to sign consent and did so on behalf of their child.

Two hundred and twenty-seven children and their parents were invited to take part in the study. However, due to incomplete data obtained in the questionnaires or errors in their completion, 19 of them were excluded. Finally, two hundred and eight children with AR participated in the study (89 girls and 119 boys, aged 6–17, mean age 11.7 ± 3). They were recruited from randomly selected outpatient clinics in Wroclaw, the capital of Lower Silesian voivodeship in southwest Poland, and one rural region, in the distance of around 40 km away from Wroclaw. All parents (168 mothers and 40 fathers) filled in questionnaires, designed for the purposes of this study, which included information about children’s socio-demographic characteristics, health status and ongoing treatment. The diagnosis of AR was made based on the clinical history, physical examination and positive results of skin prick tests. Skin prick tests were performed with 10 commercial extracts of inhalant allergens: grass pollens, birch pollens, mugwort, alder, hazel, common rye, cat fur, *Dermatophagoides farinae*, *Dermatophagoides pteronyssin*us, *Alternaria* (HAL Allergy, The Netherlands), and with negative (saline) and positive (histamine) control solutions. Skin prick tests were considered to be positive if they induced a wheal of mean diameter of 3 mm, or more than the response to saline. One hundred and thirty-six patients (65% of all subjects) with allergy to seasonal allergens were examined during the pollen season. The average duration of the disease since the diagnosis of AR was 2.5 years in the study group, and the average patient’s age at the time of diagnosis of AR was 9.5 years (3–17 years). In the analyses, the whole population was divided into two age groups, in accordance with the age groups proposed by the QoL questionnaires used in our study (separate for younger and older children): group 1 – children aged 6 to 13, and group 2 - children aged 14 to 17. The characteristics of the patients are presented in Table 1.

**Table 1:** Patients characteristics

Characteristic	Whole group (6–17 years) *N* = 208		Group I (6–13 years) *N* = 133		Group II (14–17 years) *N* = 75	
	*N*	%	*n*	%	*N*	%
Gender:						
Girls	89	42.8	56	42.1	33	44.0
Boys	119	57.2	77	57.9	42	56.0
Residental area:						
> 15.000 residents	76	36.5	45	33.8	31	41.3
< 15.000 residents	36	17.3	17	12.8	19	25.3
Rural	96	46.2	71	53.4	25	33.3
Mother’s education:						
< 9 years	7	3.4	4	3.0	3	4.0
10–12 years	99	47.6	55	41.4	44	58.7
> 12 years	102	49.0	74	55.6	28	37.3
Father’s education:						
< 9 years	7	3.4	4	3.1	3	4.1
10–12 years	129	63.2	81	62.3	48	64.9
> 12 years	68	33.3	45	34.6	23	31.1
Age (years):						
*M* ± *SD*	11.7 ± 3.0		9.8 ± 2.0		15.0 ± 1.3	
*Me* [*Q*_1_; *Q*_3_]	12 [9; 14]		10 [8; 12]		15 [14; 16]	
*Min* – *Max*	6–17		6–13		14–17	
Allergy to seasonal allergens	136	65.4	85	63.9	51	68.0
Allergy to perennial allergens	72	34.6	48	36.1	24	32.0
Treatment in last 2 weeks:						
Nasal corticosteroid	53	25.5	34	25.6	19	25.3
Nasal noncorticosteroid	45	21.6	32	24.1	13	17.3
Oral antihistamines	122	58.7	83	62.4	39	52.0
Symptoms present in last 2 weeks:						
Sneezing	158	76.0	103	77.4	55	73.3
Rhinorrhea	151	72.6	100	75.2	51	68.0
Nasal pruritus	124	59.6	83	62.4	41	54.7
Watery eyes	79	38.0	51	38.3	28	37.3

*M* mean, *SD* Standard deviation, *Me* Median, *Q1* Lower quartile, *Q3* Upper quartile*, Min* Minimum, *Max* Maximum

The children were asked to evaluate their rhinitis symptoms using two scales: the T4SS (*Total 4 Symptom Score)* and the VAS *(Visual Analogue Scale).* The T4SS is a subjective scoring system for the determination of symptom severity in patients with AR. The following symptoms were assessed by children: nasal obstruction, sneezing, rhinorrhoea and itchy nose. Each of these symptoms was rated on a scale of 0 to 3 points depending on the severity of a symptom. Children expressed global discomfort caused by AR symptoms in the previous week using the VAS. The scale ranged from “not at all bothersome” (0 mm) to “intolerably bothersome” (100 mm).

The QoL assessment included the KINDL-R questionnaire [10]. It comprises separate generic forms for three age groups: Kiddy-KINDL (4–6 years of age), Kid-KINDL (7–13 years of age) and Kiddo-KINDL (14–17 years of age), and a proxy version for parents. Children answered the questions independently (assisted by medical staff in case of reading difficulties); the parents assessed the QoL of their children independently. The KINDL-R questionnaire consists of 24 questions with five possible answers (1 = “never”, 2 = “rarely”, 3 = “sometimes”, 4 = “often”, 5 = “all the time”). The questions are related to the way the patients felt in the previous week, and cover issues of six dimensions of the QoL: physical well-being, emotional well-being, self-esteem, family, friends and everyday functioning (school or nursery school/kindergarten). The sub-scales of these six dimensions can be combined to produce a total score. On account of the particular difficulties associated with interviewing young children, the structure of the Kiddy-KINDL differs from that of the other questionnaires (Kid/Kiddo). The self-report version is simpler and consists of 12 items, two for each dimension. The psychometric results reveal a high degree of reliability, the KINDL internal consistency (Cronbach’s α) has been reported at ≥0.70 for the subscales and 0.80 for the total scale. Correlations with similar well-being questionnaires have shown acceptable convergent validity, and a high correlation (*r* > 0.70) with subscales of the Child Health Questionnaire, as well a satisfactory discriminant validity [11].

The mean values (M), standard deviations (SD), median (Me), lower quartiles (Q1) and upper (Q3) as well as the variability range (Min) and (Max) were calculated for all quantitative traits. The assessment of the compatibility of empirical distributions with the theoretical normal distribution, depending on the sample size, was checked by the Kolmogorow-Smirnov test or the Shapiro-Wilk test. The significance of differences in the assessment of the QoL carried out by parents and children was verified using the Wilcoxon test. To assess the significance of differences between the T4SS components, Friedman’s test was used, and Dunn’s test for multiple comparisons. The Pearson correlation coefficient (r) was used to assess the strength and direction of the correlation between QoL domains and severity of VAS and T4SS symptoms. The Spearman’s rank correlation coefficient (rho) was used to assess the correlation between VAS and T4SS. Qualitative features are presented in the tables in the form of numbers (n) and fractions (%). In all tests, the values of *p* < 0.05 were deemed to be statistically significant. The analyses were performed with STATISTICA v.12.5 (StatSoft, Inc., Tulsa, OK. USA).

## Results

**Fig. 1:**
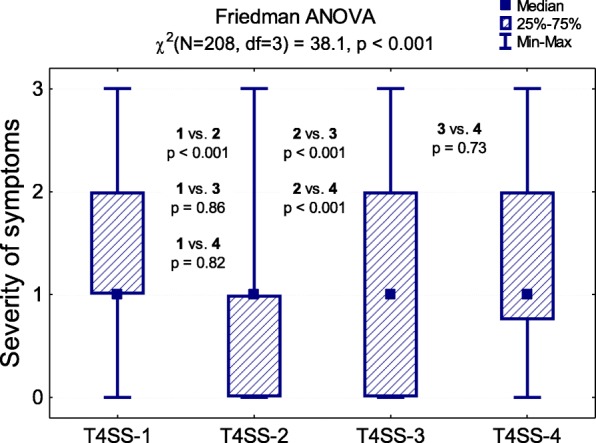
Severity of symptoms in the examined children and the Friedman test result. T4SS-1 sneezing, T4SS-2 nasal itching, T4SS-3 nasal obstruction, T4SS-4 rhinorrhea

**Fig. 2:**
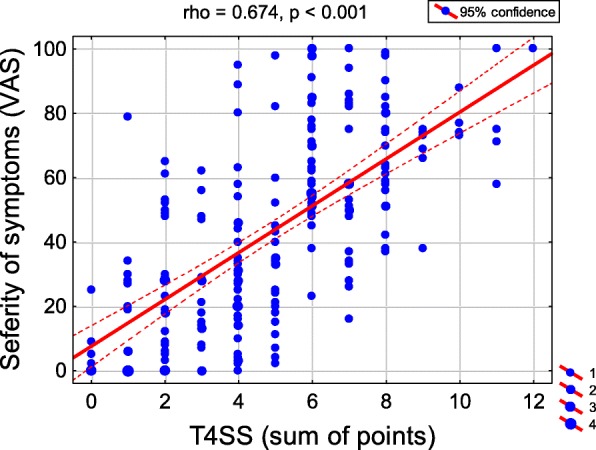
Correlation of symptom severity assessed by using VAS and T4SS scales and Spearman rank correlation coefficient (rho)

The average T4SS value was 4.9 (range 0–12). Considering the whole group, the most common response regarding the severity of each symptom - sneezing, nasal itching, nasal obstruction and rhinorrhea - was “mild severity”. Nasal itching was significantly less severe than other symptoms (*p* < 0.001), (Fig. 1). The average VAS score reported by children was 43.3 ± 28.9. A strong positive correlation was observed between the severity of symptoms reported using the VAS and the T4SS scales (rho = 0.674), (Fig. 2). There were no significant correlations between the child’s age and the severity of symptoms assessed using both scales.

The relationship between the T4SS and the VAS scale results and the socio-demographic and clinical features was verified in terms of gender, place of residence, type of AR and the number of allergens to which the child was allergic. In case of both the T4SS and VAS scale, the severity of symptoms in children with seasonal rhinitis was significantly higher than in children allergic to perennial allergens. There was no such correlation between the severity of symptoms and gender, place of residence or the number of allergens the child was allergic to.

The QoL total scores on the KINDL questionnaire were 45.6 ± 8.5 for children and 73.7 ± 10.7 for their parents. Children considered physical health to be the best domain; in the case of the age groups - 70.7 ± 18.0 for younger children, and 67.3 ± 18.0 for older children. The respondents awarded the lowest number of points to social contacts - 23.1 ± 19.3 for children aged 6–13, and 26.2 ± 18.9 for children aged 14–17. In the assessment of the children, the QoL in the “emotional well-being” domain was better in the case of the age group of 6–13 in comparison with older children (50 vs. 44 points, *p* = 0.015). According to parents, their children had the best QoL in the domain of social contacts, and the worst in the area of self-esteem and physical health. In younger children, parents awarded the highest number of points to emotional well-being and social contacts, and the lowest number of points to self-esteem.. In the group of older children, in the opinion of parents, the highest and the lowest rated domains were family contacts and physical health, respectively. The parents of younger children considered their QoL to be better than the parents of older children in the school/kindergarten domain (75 vs. 63, *p* < 0.001), and in the overall assessment (77 vs. 71; *p* = 0.024). There were no significant differences in any domains of the QoL assessed by both children and their parents in terms of the child’s gender. However, in the older age group, the parents of girls considered the QoL of their daughters to be poorer than the parents of boys did.

**Fig. 3:**
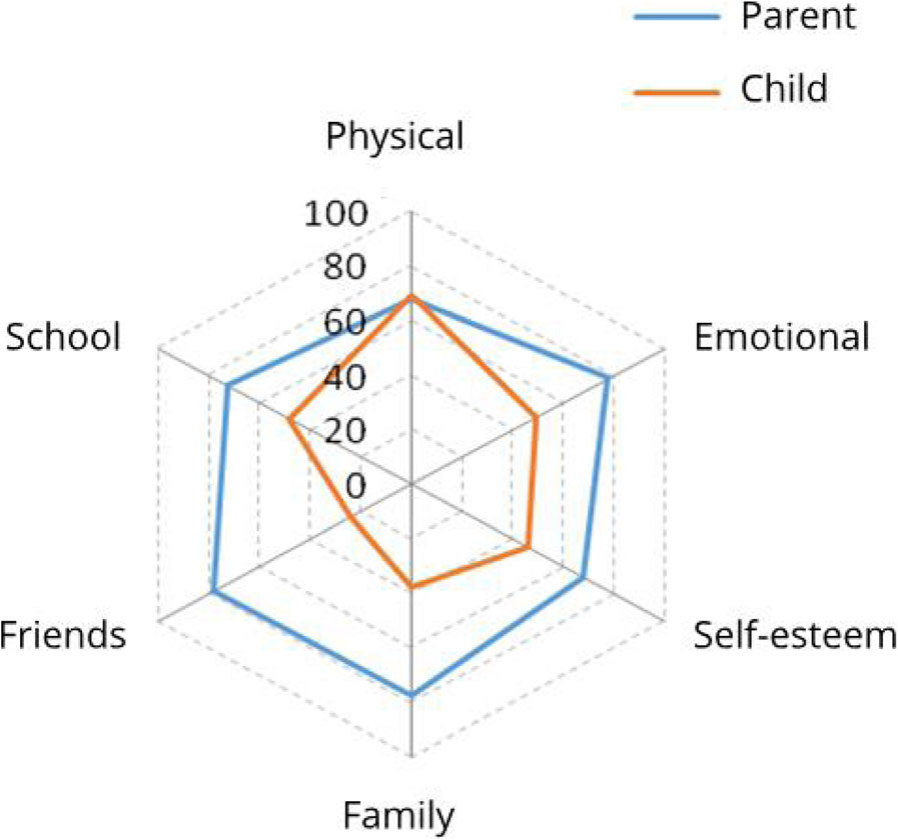
Child and parent mean scores for different life domains of children’s life on the KINDL

In all the domains, except for physical health, the QoL was rated significantly higher by parents than by children (*p* < 0.001). The biggest discrepancy occurred in the domains: social contacts and family (Table 2, Fig. 3).

**Table 2:** Child and parent assessment of child’s quality of life on the KINDL-R questionnaire

Dimensions of KINDL-R	Evaluator		Parent vs. Child *P*
	Parent	Child	
1. Physical Well-Being (points):			0.197
*M* ± *SD*	67.7 ± 17.9	69.5 ± 18.1	
*Me* [*Q*_1_; *Q*_3_]	69 [56; 81]	69 [56; 81]	
*Min* – *Max*	13–100	0–100	
2. Emotional Well-Being (points):			< 0.001
*M* ± *SD*	77.7 ± 14.4	49.4 ± 11.8	
*Me* [*Q*_1_; *Q*_3_]	81 [69; 88]	50 [44; 56]	
*Min* – *Max*	31–100	13–100	
3. Self-Esteem (points):			< 0.001
*M* ± *SD*	67.8 ± 16.3	45.9 ± 15.4	
*Me* [*Q*_1_; *Q*_3_]	69 [56; 75]	44 [38; 56]	
*Min* – *Max*	19–100	19–100	
4. Family (points):			< 0.001
*M* ± *SD*	77.3 ± 15.5	37.4 ± 14.8	
*Me* [*Q*_1_; *Q*_3_]	81 [69; 88]	31 [25; 44]	
*Min* – *Max*	19–100	13–100	
5. Friends (points):			< 0.001
*M* ± *SD*	78.0 ± 15.9	24.2 ± 19.2	
*Me* [*Q*_1_; *Q*_3_]	81 [69; 88]	19 [9; 38]	
*Min* – *Max*	13–100	0–88	
6. School/kindergarten (points):			< 0.001
*M* ± *SD*	72.8 ± 16.0	48.3 ± 16.1	
*Me* [*Q*_1_; *Q*_3_]	75 [63; 88]	44 [38; 56]	
*Min* – *Max*	19–100	13–100	
Total (points):			< 0.001
*M* ± *SD*	73.7 ± 10.7	45.6 ± 8.5	
*Me* [*Q*_1_; *Q*_3_]	75 [68; 81]	45 [41; 49]	
*Min* – *Max*	39–100	30–88

We compared the fathers’ opinions about their children’s QoL with the opinions of mothers regarding their children’s QoL. The child’s QoL was considered to be higher by fathers than by mothers, except in the domain of “school/kindergarten”. A significant difference was found in the “physical health” domain (75 vs. 69 points, *p* = 0.008), and in the overall QoL assessment (77 vs. 74 points, *p* = 0.044). The level of parents’ education did not affect the scores. However, better educated mothers assessed the QoL of their children as worse.

In children, the place of residence did not affect their QoL score. Parents living in rural areas evaluated their children’s overall QoL at a higher level than city residents did, especially in the domain of social contacts.

Considering the type of AR, it was observed that the overall QoL was rated higher by children with perennial AR than by children with seasonal AR (46 vs. 44 points; *p* = 0.043). The significant difference was seen in the “school/kindergarten” domain (50 vs. 44 points, *p* = 0.045); the child’s AR type did not have an influence on the assessment results in the parents’ opinion.

Surprisingly, the number of allergens the child was allergic to, and the kind of treatment the child received, did not affect the result of the assessment of the QoL made by both children and their parents. Only parents of children undergoing specific immunotherapy reported better QoL of their children.

There was a significant negative correlation between the results of the VAS and the T4SS scale of AR symptoms, and the assessment of the QoL by children in the domain of “physical health”. In the parents’ assessment, such a negative correlation was found for the VAS scoring and the “physical health” domain, as well as for the T4SS scale and the “physical health”, “emotional well-being”, and “social contacts” domain, and the total subjective assessment of the QoL (Table 3).

**Table 3:** Values of linear correlation coefficients (r) VAS, T4SS with the assessment of the quality of life made by parent and child

Dimensions of KINDL-R	Parent		Child	
	VAS	T4SS	VAS	T4SS
Physical Well-Being	**−0.273** (−0.394 to − 0.142)	**− 0.277** (− 0.398 to − 0.146)	**−0.259** (− 0.382 to − 0.128)	**−0.326** (− 0.442 to − 0.198)
Emotional Well-Being	−0.109 (− 0.242 to 0.027)	**−0.177** (− 0.306 to − 0.042)	−0.043 (− 0.178 to 0.093)	0.024 (− 0.113 to 0.159)
Self-Esteem	−0.044 (− 0.179 to 0.093)	−0.062 (− 0.196 to 0.075)	0.066 (− 0.070 to 0.200)	0.080 (− 0.057 to 0.214)
Family	0.097 (− 0.039 to 0.230)	−0.056 (− 0.191 to 0.081)	−0.073 (− 0.207 to 0.064)	0.039 (− 0.098 to 0.174)
Friends	−0.041 (− 0.176 to 0.095)	**−0.165** (− 0.295 to − 0.030)	0.096 (− 0.040 to 0.229)	**0.188** (0.053 to 0.316)
School/kindergarten	−0.040 (− 0.175 to 0.097)	−0.056 (− 0.191 to 0.080)	−0.052 (− 0.187 to 0.085)	−0.059 (− 0.193 to 0.078)
TOTAL	− 0.125 (− 0.256 to 0.012)	**−0.201** (− 0.328 to − 0.066)	−0.052 (− 0.187 to 0.084)	−0.023 (− 0.158 to 0.113)

## Discussion

The main findings of our study are as follows: allergic rhinitis impaired the QoL in the studied population. In the children’s opinion, social contacts were impaired the most. The score of the emotional domain of the QoL questionnaire was lower in older children. The parents assessed the QoL as much better than their children did. In the parents’ but not children’s opinion, girls in the older age group reported a poorer QoL than boys. Fathers tended to evaluate their children’s QoL higher than mothers did. The educational level of parents did not affect this assessment, but better educated mothers considered the QoL of their children to be slightly lower. The patients with allergy to perennial allergens had a better QoL compared to those allergic to seasonal allergens. In the children’s opinion the place of residence did not affect their QoL. The children’s QoL was rated higher by parents living in the country than by city residents.

In the case of physical health, the QoL of children correlates with the severity of the disease symptoms measured using the VAS and the T4SS scales.

It has been shown that the symptoms of AR have a negative impact on the QoL of the child and the child’s subjective perception, assessed using the QoL questionnaires, provides a more realistic description of it. In a reference data from Germany, children’s QoL reported by their parents was 76.3 points which is almost identical to our study [12]. In a citied research the 11- to 17-year-old children had the opportunity to report their QoL on their own, filling the KINDL-R questionnaire. Their total score of KINDL was 73.0 points. This is a significantly different result from ours (45.6 total score) which leads to the conclusion that AR decreases QoL. This comparison also shows the importance of asking children themselves about their QoL rather than relying only on parental opinion.

Children with AR may be less willing to participate in various types of activities - physical activity (e.g. sports), social activities (meetings with friends, spending time together), as well as formal and informal activities [13]. Nasal obstruction and sleeping disorders caused by AR may decrease their ability to learn and focus.

In our study, younger children rated their QoL at the level of 45.6 ± 8.5 points, i.e. slightly higher than older children, especially in the case of emotional well-being. A recent study carried out in relation to 1248 children, aged 7–17, compared the self-assessed general QoL using KINDL-R in 514 children with congenital heart disease and 734 healthy ones. The QoL results were higher than those obtained in our study: 78.6 ± 9.8 for children with heart problems, and 75.6 ± 10.1 for healthy ones [14]. KINDL-R was also used to assess the QoL of younger children than those participating in our study - Villalonga-Olives et al. showed the following average results of KINDL-R on the basis of a group of 317 pre-schoolers: 67.88 ± 17.3 for boys, and 71.70 ± 16.37 for girls [15]. The fact that younger children have a better QoL was also shown in a study conducted on children aged 8–18, in 12 European countries, including Poland, which assessed the QoL using the KIDSCREEN-52 questionnaire [16]. Dynamic changes in the perception of the outside world and of oneself, depending on age, make children as a whole group inconsistent for the QoL research. Parents assessed that their children had the best QoL in the domain of social contacts, and the worst in the domain of self-assessment and physical health. In every domain, except for physical health, the children’s QoL was rated higher by parents than by the children themselves. The biggest discrepancies concerned social contacts and family. Presumably, parents noticed the impact of the disease on the child’s well-being and physical health, but underestimated its importance for other aspects of life. The QoL of children was better in the opinion of fathers than in the opinion of mothers. It may reflect differences in emotional involvement and parental feelings related to the child’s health situation. In our study, the QoL was rated higher by the parents of younger children than by the parents of older children.

In the group of healthy children, Meyer et al. also obtained higher, yet similar results in the assessment of the QoL by parents to those provided by children (the KINDL-R results:: 77.1 ± 9.5 and 78.1 ± 9.4, respectively). The highest differences in the assessment were related to the area of self-assessment and emotional well-being: in these two domains the children’s QoL was rated higher by parents than by children themselves [17]. In our study, the QoL of the child assessed by the parent in the group of younger children did not depend on the child’s gender. In the case of older children, parents observed a poorer QoL in the case of girls. In a Norwegian study using KINDL-R, the QoL, assessed by 1743 children and at least one parent of each child, was compared. Apart from the “family” domain, parents’ rating was significantly higher than that of their children. The gender of the child, and whether the answers regarding the child were given by his/her mother or father were irrelevant [7]. According to the literature, parents of chronically ill children underestimate their QoL, and parents of healthy ones overestimate their well-being [18]. In addition, parents provided more consistent information on children under the age of 12, and less consistent information regarding teenagers [19]. One could assume that the parents of children examined in our study do not consider AR to be a serious disease.

In our study, the place of residence did not influence the assessment of the QoL in children. However, in the case of children living in rural areas, parents assessed that children had a better QoL, especially in the domain of social contacts. Perhaps this was due to the fact that in villages inhabited by smaller populations, social contacts are better, people are not anonymous, and therefore people maintain better interpersonal relationships. In other studies, children from rural areas had a higher QoL compared to their peers living in cities. The authors partially associated this fact with the protective effect of the rural lifestyle on the prevalence of allergic diseases. The biggest differences were observed in the domain of physical health [8, 9].

In our study, the number of sensitizing allergens did not affect the assessment of the QoL. Children with AR who were allergic to seasonal allergens had poorer QoL compared to children with perennial AR. Different observations were made by Chen et al. [20]. The differences may occur due to the fact that we examined children during the pollen season when their symptoms were most severe, and in the quoted study the results were collected during the off-pollen season.

Our study showed a relationship between the results obtained using the VAS and the T4SS scales, which is consistent with the previous data found in the literature [21, 22]. Children allergic to seasonal allergens had higher T4SS and VAS scores compared to patients allergic to perennial allergens. A negative correlation was found between the severity of symptoms measured using the T4SS scale and the QoL in the domain of physical health. The more severe the symptoms, the worse the results of the assessment of the QoL by children. From the parents’ point of view, the increase in the symptoms has a negative impact on the overall perception of their child’s QoL, especially on physical health, emotional well-being and social contacts. These results are also consistent with the previous research - Ciprandi et al. observed a correlation between the QoL and the severity of symptoms while examining 123 young adults. The more points on the T4SS scale, the lower the QoL measured using the specific questionnaire [23]. We observed that children with a higher VAS score considered their QoL to be poorer in the physical health domain. The parents’ opinion was the same. These results comply with the literature, even though different authors used different questionnaires to assess the QoL [22, 24].

Some of the limitations of our study are presented below. It may seem that the use of a generic questionnaire, such as KINDL-R, will result in a lower sensitivity of the QoL assessment. This questionnaire was successfully used in many other studies, not only involving healthy children. It should also be noted that the most popular questionnaire specific to AR – PRQLQ - for younger children is not available in Polish. An attempt was made to contact the creator of the questionnaire - Professor Juniper and the MAPI Institute which is responsible for the translation of these surveys into other languages. Due to complicated procedures, the consent authorising the translation of the questionnaire was not given.

Moreover, the study group was not uniform in terms of taking medication. It would have been perfect if all the patients had discontinued treatment for a specific period before the study, but because of severe symptoms of AR, it was not always possible. Nevertheless, it did not affect the assessment of the child’s QoL. On the other hand, according to our knowledge, our study was the first one in Poland in which the QoL in children was evaluated such precisely. In our study the QoL in children with AR was evaluated by comparing the answers given by children and their parents. Furthermore, a comparison of QoL and the severity of symptoms was made. The QoL of children is rarely assessed by children themselves.

## Conclusions

In conclusion, our findings indicated that AR can disrupt the QoL. Assessing the QoL in children is an important aspect to consider while managing patients with AR. Parents tend to overestimate their children’s QoL comparing to the children’s own assessment. Taking that difference in assessment into account, it is worth using questionnaires that give the opportunity to examine both the child and the parent. The QoL in children with AR correlated with the severity of clinical symptoms of the disease. The place of residence had no influence on the QoL of children with allergic rhinitis.

Careful observation of the reported symptoms, proper diagnosis, and well-chosen treatment may significantly improve the comfort of the children and help them to reduce the limitations in everyday activities, school, as well as relationships with their family and friends. Evaluation of the QoL in children is an essential issue in clinical investigation of patients with AR. It is of great importance to ask children themselves about their QoL than rely only on parental opinion.

## Data Availability

The datasets used and/or analysed during the current study are available from the corresponding author on reasonable request.

## Abbreviations

AR: Allergic rhinitis
ECAP: Epidemiology of Allergic Diseases in Poland
KINDL-R: The Revised Children Quality of Life-Questionnaire
QoL: Quality of life
T4SS: Total 4 Symptom Score
VAS: Visual Analogue Scale
